# Indoleamine 2,3-dioxygenase 1 (IDO1) activity in leukemia blasts correlates with poor outcome in childhood acute myeloid leukemia

**DOI:** 10.18632/oncotarget.1504

**Published:** 2013-10-30

**Authors:** Valentina Folgiero, Bianca M. Goffredo, Perla Filippini, Riccardo Masetti, Giuseppina Bonanno, Roberta Caruso, Valentina Bertaina, Angela Mastronuzzi, Stefania Gaspari, Marco Zecca, Giovanni F. Torelli, Anna M. Testi, Andrea Pession, Franco Locatelli, Sergio Rutella

**Affiliations:** ^1^ Department of Pediatric Hematology/Oncology and Transfusion Medicine, IRCCS Bambino Gesù Children's Hospital, Rome, Italy; ^2^ Department of Laboratory Medicine, IRCCS Bambino Gesù Children's Hospital, Rome, Italy; ^3^ Department of Pediatrics, S. Orsola-Malpighi Hospital, Bologna, Italy; ^4^ Department of Gynecology and Obstetrics, Catholic University Med. School, Rome, Italy; ^5^ Department of Pediatric Hematology/Oncology, IRCCS Fondazione Policlinico San Matteo, Pavia, Italy; ^6^ Department of Cellular Biotechnologies and Hematology, Sapienza University, Rome, Italy; ^7^ University of Pavia, Pavia, Italy

**Keywords:** Acute myeloid leukemia, IDO1, immune escape, regulatory T cells

## Abstract

Microenvironmental factors contribute to the immune dysfunction characterizing acute myeloid leukemia (AML). Indoleamine 2,3-dioxygenase 1 (IDO1) is an interferon (IFN)-γ-inducible enzyme that degrades tryptophan into kynurenine, which, in turn, inhibits effector T cells and promotes regulatory T-cell (Treg) differentiation. It is presently unknown whether childhood AML cells express IDO1 and whether IDO1 activity correlates with patient outcome.

We investigated IDO1 expression and function in 37 children with newly diagnosed AML other than acute promyelocytic leukemia. Blast cells were cultured with exogenous IFN-γ for 24 hours, followed by the measurement of kynurenine production and tryptophan consumption. No constitutive expression of IDO1 protein was detected in blast cells from the 37 AML samples herein tested. Conversely, 19 out of 37 (51%) AML samples up-regulated functional IDO1 protein in response to IFN-γ. The inability to express IDO1 by the remaining 18 AML samples was not apparently due to a defective IFN-γ signaling circuitry, as suggested by the measurement of signal transducer and activator of transcription 3 (STAT3) phosphorylation. Co-immunoprecipitation assays indicated the occurrence of physical interactions between STAT3 and IDO1 in AML blasts. In line with this finding, STAT3 inhibitors abrogated IDO1 function in AML blasts. Interestingly, levels of IFN-γ were significantly higher in the bone marrow fluid of IDO-expressing compared with IDO-nonexpressing AMLs. In mixed tumor lymphocyte cultures (MTLC), IDO-expressing AML blasts blunted the ability of allogeneic *naïve* T cells to produce IFN-γ and promoted Treg differentiation. From a clinical perspective, the 8-year event-free survival was significantly worse in IDO-expressing children (16.4%, SE 9.8) as compared with IDO-nonexpressing ones (48.0%, SE 12.1; *p*=0.035).

These data indicate that IDO1 expression by leukemia blasts negatively affects the prognosis of childhood AML. Moreover, they speak in favor of the hypothesis that IDO can be targeted, in adjunct to current chemotherapy approaches, to improve the clinical outcome of children with AML.

## INTRODUCTION

Childhood acute myeloid leukemia (AML) is a rare and heterogeneous disease, with an annual incidence of 7 cases per million children younger than 15 years [1]. Patients' stratification according to biological characteristics and response to induction therapy, together with the intensification of conventional chemotherapy and advent of better supportive care strategies, have improved prognosis, the current 5-year overall survival (OS) rates being in the order of 70% [1]. However, 5% of children with AML have refractory disease and 30% still experience relapse. Allogeneic hematopoietic stem cell transplantation (HSCT) can be curative for some patients with relapsed AML, who are successfully given re-induction chemotherapy [1, 2]. The major therapeutic benefit of allogeneic HSCT results from the so-called graft-versus-leukemia (GVL) effect, namely, the recognition by donor-derived T cells of antigens expressed on host malignant cells. The existence of a GVL effect is underpinned by the evidence that infusion of donor lymphocytes can induce disease remission in patients who have relapsed following allogeneic HSCT [3, 4]. These findings imply that the stimulation of anti-leukemia immunity may be crucial to improve the clinical outcome of patients with AML.

Escape from immunosurveillance through immunoselection, also known as immunoediting, and immunosubversion, i.e., active suppression of the immune response, is a hallmark of cancer [5]. The immune evasion mechanisms exploited by AML have begun to be elucidated but recently, and represent a major hurdle to the delivery of effective immunotherapy. AML is a systemic disease, and the microenvironment shaped by AML cells may differ in many aspects from that of anatomically-localized, solid malignancies. However, some immune escape pathways promoted by AML overlap with those fostered by solid tumors, such as expansion of naturally occurring CD4^+^FoxP3^+^ regulatory T cells (Treg) [6], expression of negative regulatory receptors [7], deletional T-cell tolerance [8] and defective T-cell co-stimulation [9].

Indoleamine 2,3-dioxygenase 1 (IDO1) is a tryptophan-catabolizing enzyme encoded by the *IDO1* gene. IDO1 oxidizes tryptophan into *N*-formylkynurenine, which is rapidly converted to kynurenine by kynurenine formamidase [10]. The IDO1-driven production of kynurenine promotes the development, stabilization and activation of Treg cells, while suppressing effector T cells, all of which contribute to immune system impairment in cancer-bearing individuals [11]. In humans, IDO1 was found to be expressed by a unique subset of dendritic cells (DC) [12] and by a variety of solid tumors, such as colorectal cancer, melanoma and serous ovarian cancer [13-16]. More recently, a dual regulatory function of IDO as a catalyst and a signaling protein has been unraveled in tolerogenic DC [17].

The expression and function of IDO1 in childhood AML have not been previously investigated. Herein, we provide evidence that IDO1 is induced in leukemia cells by IFN-γ in around half of children with AML in a signal transducer and activator of transcription 3 (STAT3)-dependent manner. IDO-expressing leukemia blasts restrain IFN-γ production by allogeneic T cells and promote the *in vitro* differentiation of *bona fide* Treg cells. From a clinical standpoint, IDO1 expression is associated with a significantly worse probability of event-free survival (EFS).

## RESULTS

### Expression of IDO1 in AML blasts

**Figure 1:**
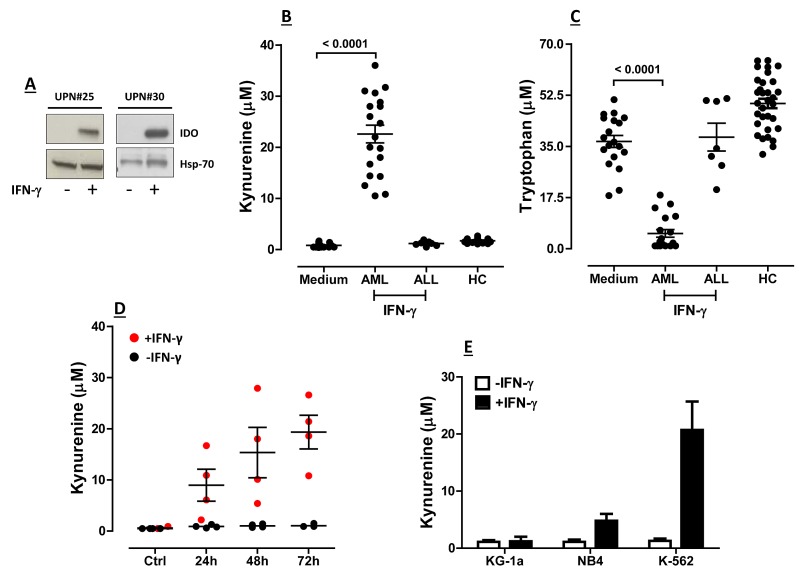
Expression and Function of IDO1 in BM Samples from Children with AML BM samples from children with AML obtained at diagnosis were cryopreserved. After thawing, BM mononuclear cells (MNC) were either stimulated with 100 ng/ml IFN-γ for up to 72 hours or were maintained in culture medium alone. Supernatants were collected and used for the measurement of kynurenine and tryptophan levels by RP-HPLC. (A) Up-regulation of IDO protein by IFN-γ (+) in 2 representative AML samples; UPN = Unique Patient Number; Hsp-70 = heat shock protein-70; (B) Release of kynurenine and (C) Consumption of tryptophan by AML blasts maintained in culture for 72 hours with or without exogenous IFN-γ. Blasts from a cohort of 7 children with acute lymphoblastic leukemia (ALL) were also challenged with IFN-γ to detect any IDO-mediated tryptophan breakdown. Comparisons between groups were performed with the Mann-Whitney U test for paired determinations. HC = healthy controls. Medium = blast cells maintained with complete culture medium alone; (D) Time-course experiments with AML blasts from 4 randomly selected BM samples that were either activated with IFN-γ (red dots) or left untouched (black dots). Bars depict the median and interquartile range; (E) Commercially available AML cell lines (see main text for details) were either stimulated with IFN-γ for 72 hours (black columns) or were maintained in culture medium alone (empty columns), prior to HPLC studies. Bars are representative of mean values and standard deviation recorded in 3 independent experiments run in duplicate.

Thirty-seven children with AML (median age 12 years, range 0.2-23) were retrospectively analyzed for IDO status and clinical outcome. Patients' demographics are summarized in Table I. Among the 37 BM samples analyzed in this retrospective study, 12 were from children with FAB-M1/M2 AML, 9 from children with FAB-M4 AML, 13 from children with FAB-M5 AML and 1 from a child with undifferentiated AML. Details on the FAB subgroup were unavailable in 2 children. We initially evaluated IDO1 protein levels in leukemia blasts that were either maintained in culture medium alone or were challenged with IFN-γ for 72 hours. Leukemia cells did not express IDO1 constitutively in any BM sample tested (Figure 1A), and their basal production of kynurenine was barely detectable (data not shown). Overall, treatment with IFN-γ for 72 hours translated into the up-regulation of functional IDO1 (Figure 1A) and into the long-term maintenance of IDO enzyme activity in 51% of AML cases, as reflected by heightened production of kynurenine (median 22.05 μM/L, range 6.0-36.0, in IFN-γ-stimulated cultures compared with 0.85 μM/L, range 0.4-1.7, in unstimulated cultures; *p*<0.0001) and by concomitant depletion of tryptophan (median 4.6 μM, range 1-22.6, in IFN-γ-stimulated cultures compared with 39.1 μM, range 18.2-51.0 in unstimulated cultures; *p*<0.0001; Figure 1B and 1C). Figure 1B and 1C also illustrates kynurenine and tryptophan levels in serum samples from healthy controls. Time-course experiments indicated that kynurenine release from IFN-γ-activated AML blasts progressively increased over time, reaching maximum levels 48-72 hours after the addition of IFN-γ to the cultures (Figure 1D). For the purpose of comparison, the release of kynurenine from a panel of commercially available AML cell lines is shown in Figure 1E, both constitutively and after challenge with IFN-γ. Regrettably, the retrospective nature of our study precluded the measurement of serum kynurenine and tryptophan, an estimate of IDO systemic activity, in the 37 children with AML that we enrolled [18, 19].

**Table I:** Patients' demographics and clinical characteristics

Patient	Karyotype/Molecular lesion(s)	FAB subgroup	Age at diagnosis	IDO1 status	CR	Relapse	Alive
UPN #1	Normal	M2	13.0	Negative	Yes	No	Yes
UPN #2	t(8;21) (q22;q22)	M2	13.0	Negative	Yes	No	Yes
UPN #3	Normal	M1	10.0	Negative	Yes	No	Yes
UPN #4	Normal	M2	4.9	Negative	Yes	No	Yes
UPN #5	Normal	M5	12.0	Negative	Yes	No	Yes
UPN #6	Normal	M1	3.0	Negative	Yes	No	Yes
UPN #7	t(8;21) (q22;q22)	M2	9.0	Negative	Yes	No	Yes
UPN #8	Normal	M1/M2	1.0	Negative	Yes	No	Yes
UPN #9	Normal	M2	15.0	Negative	Yes	No	Yes
UPN #10	N.A.	M1	15.4	Negative	No	Yes	No
UPN #11	Normal	N.A.	N.A.	Negative	No	Yes	No
UPN #12	FLT3-ITD	U	6.0	Negative	No	Yes	Yes
UPN #13	Normal	M2	3.4	Negative	Yes	Yes	Yes
UPN #14	t(6;9) - FLT3-ITD	M4	23.0	Negative	Yes	Yes	No
UPN #15	Normal	M5	15.5	Negative	Yes	Yes	No
UPN #16	t(8;21) (q22;q22)	M2	11.3	Negative	Yes	Yes	Yes
UPN #17	t(8;21) (q22;q22)	M2	9.0	Negative	Yes	Yes	No
UPN #18	FLT3-ITD	N.A.	14.0	Negative	N.A.	Yes	No
UPN #19	t(9;11)(p22;q23) - MLL-AF9	M5	1.0	Positive	No	No	No
UPN #20	t(10;11) - MLL-AF10	M5	6.0	Positive	Yes	No	Yes
UPN #21	inv(16)(p13q22)	M4	16.0	Positive	Yes	No	No
UPN #22	t(8;21) (q22;q22)	M4	16.0	Positive	Yes	No	Yes
UPN #23	t(10;11) - MLL-AF10	M4/M5	4.0	Positive	Yes	No	Yes
UPN #24	FLT3-ITD	M4	12.3	Positive	Yes	No	Yes
UPN #25	inv(16) (p13q22)	M4	17.2	Positive	Yes	No	Yes
UPN #26	t(1;11) - MLL-AF1	M5	0.2	Positive	Yes	No	Yes
UPN #27	inv(16)(p13q22)	M5	9.0	Positive	Yes	No	Yes
UPN #28	Normal	M5	16.5	Positive	N.A.	No	No
UPN #29	FLT3-ITD	M5	7.0	Positive	No	Yes	No
UPN #30	Normal	M5	14.1	Positive	Yes	Yes	No
UPN #31	FLT3-ITD	M5	15.0	Positive	Yes	Yes	No
UPN #32	Normal	M5	0.4	Positive	Yes	Yes	Yes
UPN #33	FLT3-ITD	M5	12.3	Positive	Yes	Yes	No
UPN #34	inv(16) (p13q22)	M4	13.0	Positive	Yes	Yes	Yes
UPN #35	t(10;11) - MLL-AF10	M5	4.0	Positive	Yes	Yes	No
UPN #36	inv(16) (p13q22)	M4	0.8	Positive	Yes	Yes	Yes
UPN #37	FLT3-ITD	M4	15.7	Positive	N.A.	Yes	No

Legend: UPN = unique patient number; U = undifferentiated AML; N.A. = not available; CR = complete remission; ITD = internal tandem duplication; FAB = French-American-British.

We also asked whether blast cells from acute lymphoblastic leukemia (ALL) of either B-cell or T-cell lineage would express IDO1 in either a constitutive and/or an inducible manner. However, we were unable to detect IDO protein and/or kynurenine production and tryptophan consumption by any ALL sample tested, either at baseline or after *in vitro* stimulation with IFN-γ (Figure 1B and 1C), in spite of the ability of IFN-γ to up-regulate phosphorylated STAT3 (data not shown).

Interestingly, the IDO-expressing AML cases (n=19) were assigned to either the FAB-M4 (8 out of 19 cases, 42%) or the FAB-M5 subgroup (11 out of 19 cases, 58%). By contrast, no AML sample of the FAB-M1 and FAB-M2 subgroups up-regulated IDO1 in response to IFN-γ. Cytogenetics data were available for 36 out of the 37 children enrolled in this study (Table II). Among the 13 patients with cytogenetically normal (CN) leukemia, only 3 children (23%) were classified as IDO-positive. By contrast, 5 AML cases harboring *MLL* re-arrangements and 5 AML cases with inv(16) displayed a FAB-M4/M5 morphology and up-regulated IDO1 upon treatment with IFN-γ (*p*=0.0065 by the Fisher's exact test, compared with children with CN leukemia). Among the 5 AML cases carrying t(8;21), 4 were IDO-negative. Finally, FLT3 internal tandem duplication (ITD), an independent prognostic factor for relapse in childhood AML [20], was detected in 8 children; leukemia cells of 5 of them (62%) were IDO-positive. Collectively, these data show that IDO expression segregates within AML cases of myelo-monocytic origin and is not apparently associated with a specific genetic lesion.

**Table II:** Patients' cytogenetics

	# of pts	IDO-positive	IDO-negative
Normal karyotype	13	3	10*
FLT3-ITD	8	5	3
inv(16)	5	5	0
MLL rearrangement	5	5	0
t(8;21)	5	1	4

* p=0.0065 compared with children harboring inv(16) or MLL rearrangement.

### Regulation of IDO1 expression in blasts from childhood AML

We have previously shown that microenvironmental levels of hepatocyte growth factor (HGF), a pro-angiogenic and immune-modulating cytokine, correlate with the expression of functional IDO1 in a subset of patients with multiple myeloma, a plasma cell malignancy that accounts for approximately 13% of hematological cancers in adults and has a unique ability to subvert and/or escape the anti-tumor immune response [19, 21]. HGF is also expressed by half of AML cases, inducing autocrine activation of MET receptor [22]. Also, myeloid-derived suppressor cells isolated from patients with breast cancer express IDO1 in a STAT3-dependent manner [23].

**Figure 2:**
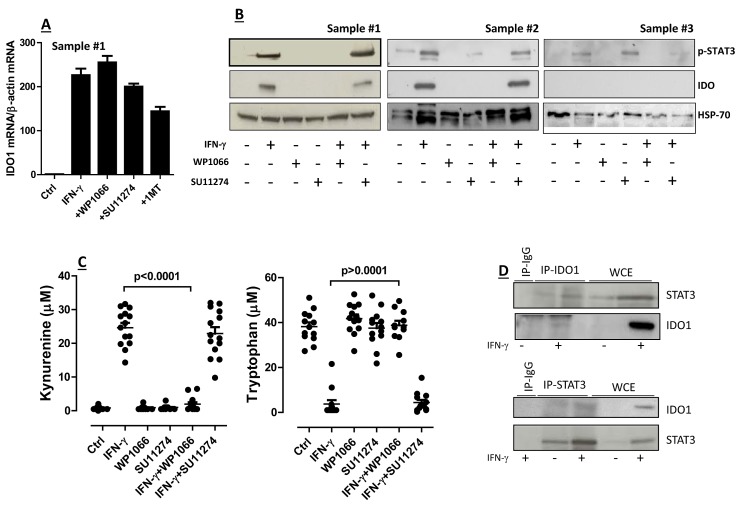
Regulation of IDO1 in AML Blasts (A) Quantitative RT-PCR and (B) Western blotting studies to detect IDO mRNA and protein in primary AML blasts. Samples were pre-treated for 2 hours with either of the following compounds: WP1066 or STAT3 Inhibitor III (28 μM final concentration), 1-methyl-tryptophan (1MT; 200 μM final concentration), SU11274 MET inhibitor (100 nM final concentration), followed by PCR or protein studies. Bars in panel A are representative of the mean ± standard deviation recorded in 3 independent experiments run in duplicate; (C) Release of kynurenine and depletion of tryptophan in supernatants of AML blasts that were pre-treated with either STAT3 inhibitors (WP1066) or MET inhibitors (SU11274) for 2 hours, and then activated with IFN-γ. Control cultures were established with either STAT3 inhibitors alone or with MET inhibitors alone. Comparisons were performed with the Mann-Whitney U test for paired determinations; (D) After challenge with IFN-γ (+) for 16 hours, leukemia cells were lysed, immunoprecipitated with anti-STAT3 antibodies and immunoblotted with anti-IDO1 antibodies (upper panel), or they were immunoprecipitated (IP) with anti-IDO1 antibodies and then immunoblotted with anti-STAT3 antibodies (lower panel). The specificity of each antibody used in these assays was confirmed using normal rabbit IgG as a negative control. Western blot runs were also performed with whole cell extracts (WCE).

With this rationale in mind, we aimed at getting insights into the molecular mechanisms potentially implicated in the regulation of IDO1 expression by AML blasts. To accomplish this, we pre-treated AML cells with either SU11274, a MET inhibitor [19], or WP1066 (STAT3 Inhibitor III) before their *in vitro* stimulation with IFN-γ. As shown in Figure 2A, IDO mRNA levels increased in IFN-γ-challenged AML blasts compared with control cultures maintained in the absence of any cytokine stimulus, and were unaffected by either STAT3 or MET inhibition. In these experiments, 1-methyl-tryptophan (1MT), the lead IDO inhibitor compound [24, 25], was used as reference. The addition of IFN-γ to leukemia blasts also translated into the up-regulation of phosphorylated STAT3 (Figure 2B), a phenomenon that was paralleled by the increase of IDO1 protein expression (see also Figures 1B and 1C). A representative Western blot experiment with IDO-expressing and IDO-nonexpressing AML samples is depicted in Figure 2B. Interestingly, WP1066, but not the MET inhibitor SU11274, abrogated the up-regulation of IDO1 protein that we observed in response to IFN-γ (Figure 2B). In line with this, kynurenine production was almost completely hampered in AML blast cells that were pre-treated with STAT3, but not with MET inhibitors (Figure 2C). The observation that IFN-γ induced the up-regulation of phosphorylated STAT3 even in IDO-nonexpressing AML cells (Figure 2B) implies that failure to up-regulate IDO1 by these samples was unlikely to be related to a defective IFN-γ signaling circuitry.

In a further set of experiments aimed at assessing the possible occurrence of physical interactions between IDO1 and STAT3, immunoprecipitates from AML blasts were subjected to Western blot runs using IDO1/STAT3-specific antibodies. The amount of IDO in whole-cell extracts (WCE) was determined by immunoblot analysis with anti-IDO before immunoprecipitation. As shown in Figure 2D, IDO1 and STAT3 proteins were barely detectable in immunoprecipitates obtained from lysates of AML blasts in the absence of IFN-γ treatment. By contrast, IDO1 was up-regulated and coprecipitated with STAT3 in AML cells activated with IFN-γ. Taken together, these data suggest that STAT3 and IDO1 are interacting partners in blasts from childhood AML.

### Microenvironmental production of IFN-γ in childhood AML

We reasoned that IFN-γ may be the primary cytokine stimulus driving *in vivo* IDO expression in childhood AML, in analogy with other normal and tumor cell types [26] and also based on previously published data with human AML cell lines [27]. Also, it is conceivable that IDO^−^ AML blasts may start expressing IDO when exposed to an IFN-γ-rich inflammatory *milieu*, implying that *in vivo* immune resistance mechanisms centered on IDO could be the result of AML blast cell interactions with IFN-γ-producing cell types, such as T cells or NK cells.

**Figure 3:**
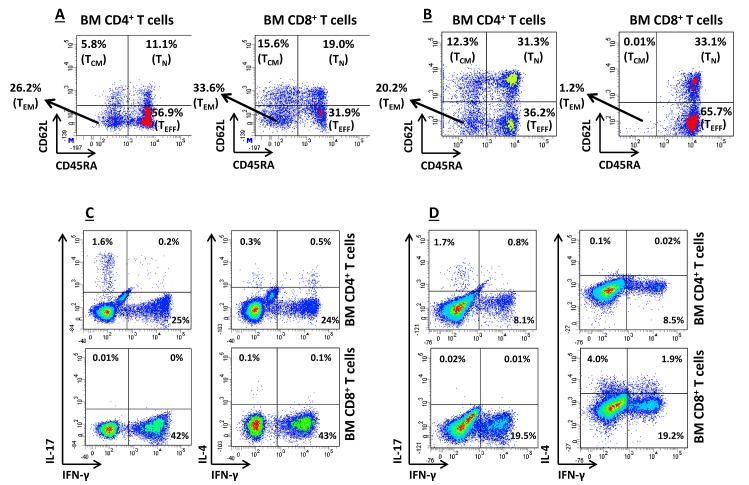
In vitro T-cell Production of IFN-γ (A) and (B) BM-resident T cells were classified as effector memory (T_EM_), central memory (C_M_), naïve (N) or effector (T_EFF_) T cells based on the expression pattern of CD45RA and CD62L, as previously reported [36]. Antigen expression patterns in two representative BM samples from children with IDO-expressing AML are shown. BM MNC were first gated on CD4^+^ or CD8 T cells, followed by the quantification of CD62L/CD45RA expression. Quadrant markers were set according to the proper isotypic control; (C) and (D) Production of IFN-γ at the single-cell level in 2 representative BM samples from IDO-positive AMLs. Details on culture conditions were provided in materials and Methods. BM MNC were first gated on CD4^+^ or CD8^+^ T cells, followed by the quantification of IFN-γ, IL-4 and IL-17 expression. Markers were set according to the proper isotypic control. The percentage of cells staining positively for a given antigen is indicated.

We initially measured IFN-γ levels in the BM fluid of a subgroup of 11 randomly selected children with AML, for whom sufficient amounts of surplus diagnostic material were available. IFN-γ levels in the BM of IDO-expressing AML cases (n=7; median 3.9 ng/ml, range 0.72-8.74) exceeded those measured in IDO-nonexpressing AML samples (n=4; median 0.97 ng/ml, range 0.8-3.68; p=0.047) and in BM blasts from a cohort of 7 children with ALL, used as controls (median 1.32 ng/ml, range 0.09-2.05; p=0.039). We then investigated the propensity of T cells infiltrating the BM of children with AML to release IFN-γ in response to polyclonal activation. As exemplified in Figure 3A-B, intra-tumoral CD4^+^ and CD8^+^ T cells mainly comprised effector memory (CD45RA^−^CD62L^−^) and effector T cells (CD45RA^+^CD62L^−^). Figure 3C-D shows that a median of 16.5% CD4^+^ and 31.2% CD8^+^ BM-resident T cells from AML patients produced IFN-γ, respectively, and suggests that intra-tumoral T cells can be an *in vivo* source of this cytokine in AML. By contrast, intracellular IL-4 was detected in a minute percentage of CD4^+^ T cells (median 0.2%); furthermore, the median percentage of IL-17-secreting CD4^+^ T cells was 1.65% (Figure 3C-D). Finally, we aimed at enumerating BM-resident NK cells in children with AML. However, NK cells were not abundant in the BM microenvironment (data not shown), thus precluding any robust measurement of their ability to produce IFN-γ at the single-cell level.

### Effects of IDO1-responsive AML blasts on T-cell function

**Figure 4:**
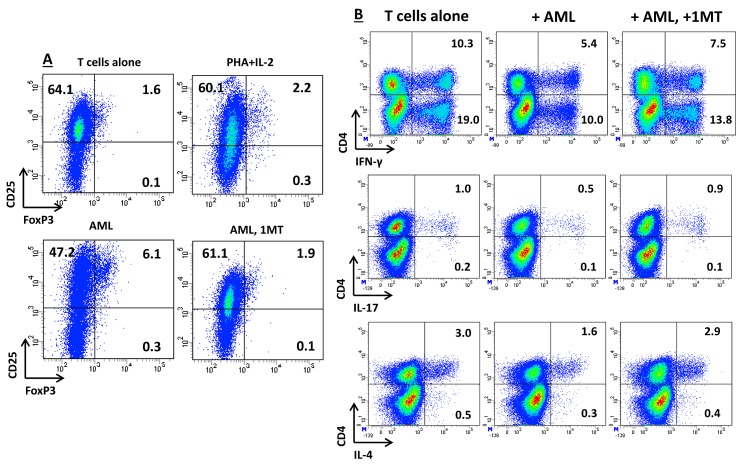
Effects of IDO-Expressing AML Blasts on T-Cell Expression of FoxP3 and on Cytokine Production Profile AML blasts were pre-treated for 2 hours with 200 μM 1MT or were left untouched, followed by stimulation with 100 ng/ml IFN-γ. IDO-expressing AML blasts were then co-cultured with allogeneic naïve CD4^+^ T cells in a standard mixed tumor lymphocyte culture (MTLC). (A) After 5 days in MTLC, cells were harvested, fixed, permeabilized and labeled with anti-CD25 and anti-FoxP3 mAbs, as detailed in Materials and Methods. Control cultures were established with CD4^+^ T cells and IL-2, either alone or combined with PHA as a polyclonal stimulus. Similar results were obtained in 3 independent experiments. The percentage of cells staining positively for a given antigen is indicated; (B) After 5 days in MTLC, T cells were activated for 6 hours with a polyclonal stimulus, consisting of PMA and ionomycin, in the presence of brefeldin-A to inhibit intracellular protein transport. Cells were then fixed, permeabilized and labeled with cytokine-specific mAbs, as detailed in Materials and Methods. The percentage of cells staining positively for a given antigen is indicated. Similar results were obtained in 3 independent experiments.

To mimica leukemia-conditioned microenvironment, allogeneic CD4^+^ T cells from healthy donors were co-cultured with AML blasts that were either pre-activated with IFN-γ or left untouched. After 5 days in mixed tumor-lymphocyte cultures (MTLC), T cells were assayed for the acquisition of FoxP3 expression and for cytokine production at the single-cell level. As shown in Figure 4A, co-culture with IFN-γ-activated AML blasts resulted into an increase of FoxP3-expressing *bona fide* Treg cells, when compared with cultures maintained in the absence of leukemia blasts or with those nurtured with a polyclonal stimulus, such as PHA, and IL-2, a Treg growth factor [28]. The differentiation of FoxP3^+^ T cells fostered by AML blasts was reverted by the addition of 1MT to the co-cultures (Figure 4A).

We also quantitated IFN-γ, IL-17 and IL-4 production at the single-cell level in T cells that were co-cultured with IDO-expressing AML blasts. As shown in Figure 4B, AML blasts inhibited IFN-γ production both by CD4^+^ and by CD8^+^ T cells. Conversely, IL-17 and IL-4 production were unaffected by IDO-expressing AML blast cells. Taken together, MTLCs indicated that IDO-expressing AML blasts favor the *in vitro* emergence of potentially leukemia-suppressive Treg cells, while restraining IFN-γ production by T cells.

### IDO expression correlates with poor survival in childhood AML

**Figure 5:**
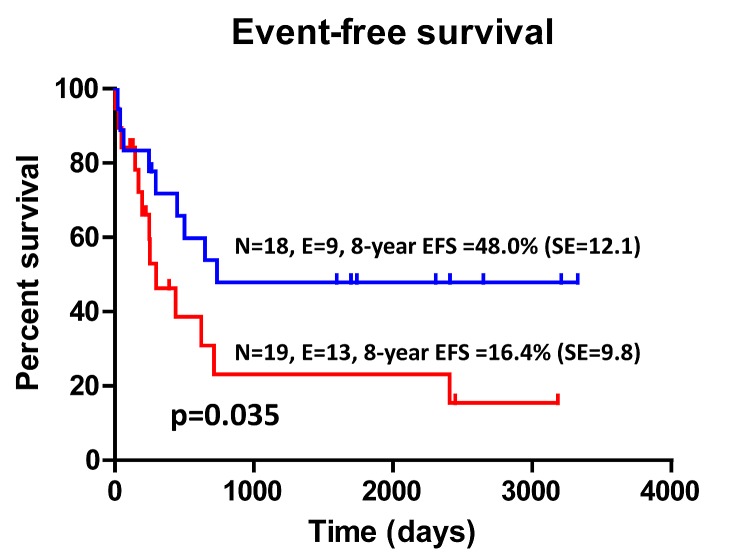
Kaplan-Meier Estimates of EFS According to IDO1 Status The 8-year EFS was calculated as detailed in Materials and Methods. Patients with AML were dichotomized based on the IFN-γ-inducible expression of IDO1. N=number; E=events; SE=standard error; EFS=event-free survival.

Data regarding the attainment of complete remission (CR) were available for 17 children in the IDO-expressing group and for 17 children in the IDO-nonexpressing group. Specifically, 15 out of 17 (88%) children with IDO-expressing AML entered into CR compared with 14 out of 17 (82%) children with IDO-nonexpressing AML (*p*=NS by the Fisher's exact test). Overall, relapse occurred in 19 children out of 37 (51%). Among the 19 children experiencing leukemia relapse, 12 (63%) were IDO-positive. In the no-relapse group (n=18), 7 children (39%) were IDO-positive. We then aimed at correlating IDO status with both disease-free survival (DFS) and overall survival (OS). With a median follow-up of 37.4 months (range 3-80), the 8-year EFS of children whose leukemia blasts up-regulated IDO1 in response to IFN-γ was equal to 16.4% (SE 9.8), compared with 48.0% (SE 12.1) in children whose AML blasts were unresponsive to IFN-γ (Figure 5; *p*=0.035). Finally, OS was 31.6% (SE 15.6) in IDO-expressing cases compared with 63.0% (SE 12.0) in children with IDO-nonexpressing AML (*p*=0.1). Collectively, these data suggest that children with IDO-expressing blasts have a worse outcome in comparison to children with IDO-nonexpressing AML.

## DISCUSSION

The microenvironment of AML is inherently suppressive for immune effector cells. Patients with AML at diagnosis have been reported to harbor dysfunctional T and NK cells, partly as a result of the tumor itself [29, 30]. In addition, AML-derived DC, AML cell lines and primary blasts from adults with AML may all express IDO1, either constitutively or after *in vitro* challenge with IFN-γ [27, 31, 32]. IDO mRNA has been detected in 52% of adults with AML in two different patient series [31, 33]. In the first one, AML blasts were found to constitutively express an active IDO protein [6, 31]. In the remaining IDO-negative cases, *in vitro* challenge with IFN-γ up-regulated IDO mRNA, but not IDO protein, suggesting that post-translational stimuli may be required for the acquisition of IDO competency by AML cells [31]. AML patients with IDO-positive blasts were shown to have a higher frequency of circulating *bona fide* CD4^+^CD25^+^ Treg cells compared with patients harboring IDO-nonexpressing leukemia blasts. *In vitro*, IDO-expressing blasts promoted the differentiation of Treg cells from naïve T cells, an effect that was abrogated by the addition of an IDO inhibitor to the co-culture. In the second study on adults with AML, IDO mRNA signals were measurable in 13 out of 25 (52%) patients [33]. In contrast with the first adult series published so far [31], all samples expressing IDO mRNA were reported to have detectable IDO activity [33].

Chamuleau and coworkers reported variable expression of IDO mRNA in primary blasts from 71 adults with AML, with no apparent correlation with cytogenetic risk profile or with FAB subtype [34]. Intriguingly, a high blast cell content of IDO mRNA was associated with lower CR rate (53% versus 84% for patients with low IDO expression), shorter relapse-free survival (6.1 months compared with 24.5 months for patients with low IDO expression) and poorer OS (7.4 months vs. 21.4 months for patients with low IDO expression) [34]. However, neither IDO protein levels nor IDO enzymatic activity could be evaluated in this study, thus precluding any definitive conclusion about the *in vivo* mechanisms than may underlie the different clinical outcome of AML patients showing high or low IDO mRNA content in BM blasts.

Another study with 184 adult patients with AML pointed out that IDO mRNA can be detected in most AML samples, irrespective of their FAB subtype [18]. Although IDO mRNA levels did not correlate with survival in this patient series, those patients with high kynurenine/tryptophan ratios (i.e., >0.075) in their serum had lower survival rates compared with patients with low kynurenine/tryptophan ratios, suggesting again that IDO activity affects clinical outcome [18]. *In vitro*, basal production of kynurenine in cultures of AML blasts obtained from a subgroup of 15 patients was low in all but 1 case, and was consistently induced by IFN-γ but not by other stimuli, such as TNF-α, PGE_2_ and LPS [18]. Taken together, the currently available studies focused on IDO in adult AML suggest that IDO is expressed by a subgroup of patients and that it may confer an adverse prognosis. However, discrepancies are still unsolved concerning the mechanisms that govern IDO expression at molecular and protein level in AML blasts.

Herein, we present the results of a multi-Centre effort aimed at investigating, for the first time, IDO1 expression and regulation in childhood AML. In our study, IDO protein and enzymatic activity were not detected constitutively but were induced in 51% of children with AML after 72-hour *in vitro* culture of primary blasts with exogenous IFN-γ. The IDO-nonexpressing AML cases were equally sensitive to IFN-γ stimulation, as suggested by the occurrence of STAT3 phosphorylation. This observation implies that IFN-γ signaling is preserved in AML cells that do not up-regulate IDO expression in response to cytokine stimulation. The ability to increase IDO1 expression was unique to AML samples, as we were unable to detect IDO protein and/or function in primary blasts from childhood ALL of either T or B-cell lineage. IDO-expressing AML samples were almost exclusively assigned to the FAB-M4/M5 subgroups. By contrast, the ability to up-regulate IDO1 in response to IFN-γ was not correlated with the cytogenetic risk profile.

Although the microenvironmental source of IFN-γ in children with AML remains to be identified, our results argue that BM-infiltrating T cells may contribute to IFN-γ production which, in turn, has the potential to drive the *in vivo* up-regulation of IDO1 in AML blasts. Our contention is backed by the higher levels of IFN-γ measured in the BM fluid of patients with IDO-expressing AML. It has been reported that IFN-γ production *in vivo* may result from the recognition of melanoma cells by autologous CD8^+^ T cells [35]. This elegant study and our own data open an intriguing scenario, whereby the immune system, rather than cancer cells themselves, may have the intrinsic ability to drive major immunosuppressive pathways. Also, intracellular IFN-γ levels in BM-resident T cells from children enrolled in our study correlated with the magnitude of the T_EM_ and T_EFF_ compartments, both of which lack CD62L expression. This observation is in agreement with previous reports showing that IFN-γ production is restricted to CD8^+^ T cells not expressing receptors that control homing to secondary lymphoid organs, such as CCR7 and/or CD62L [36].

Given the undisputed role of IDO1 in activating and stabilizing Treg cells [37], we measured intracellular FoxP3 levels after culturing allogeneic naïve T cells with IDO-expressing leukemia blasts. Notably, the frequency of *bona fide* Treg cells was higher in co-cultures established with IDO-expressing AML blasts. In line with previous findings on the ability of IDO-competent myeloma cells to tip the Th1/Th2 balance in favor of Th2 cells [19], we found that IDO-expressing AML blasts dampened the *in vitro* production of IFN-γ by T cells, an effect that was reverted by the IDO chemical inhibitor 1MT.

In conclusion, IDO1 expression is associated with poor EFS in childhood AML. Conceivably, IDO-competent AML blasts may constrain leukemia-specific immune responses *in vivo* through effects on T-cell cytokine production and on the emergence of leukemia-suppressive Treg cells. Strategies to revert immune evasion in AML with a T-cell-inflamed BM microenvironment, such as targeting of IDO1 and/or STAT3 as negative regulatory checkpoints, could be incorporated into current treatment protocols for childhood AML [38], especially in the setting of minimal residual disease.

## PATIENTS AND METHODS

### Patients' characteristics

Patients were enrolled at 4 different Clinical Centers affiliated with the ‘Italian Association of Pediatric Hematology/Oncology’ (AIEOP), and were uniformly treated according to AIEOP chemotherapy protocols [38, 39]. Surplus peripheral blood (PB) and/or BM samples cryopreserved at time of diagnosis and/or disease relapse were used for *in vitro* studies, as detailed below. Patients and/or their legal guardians gave written informed consent. The study was approved by the local Ethical Committee (protocol #714.12).

### Abs and reagents

FITC-conjugated anti-IDO antibodies (clone 700838) and recombinant human IFN-γ and IL-2 were purchased from R&D Systems (Oxon, Cambridge, UK). Rabbit anti-human IDO (H-101) and anti-IRF-1 (C-20) antibodies, and WP1066 (a STAT3 inhibitor) were purchased from Santa Cruz Biotechnology (Milan, Italy). The IDO chemical inhibitor d,l-1MT, a racemic mixture containing both the *levo* and the *dextro* isomer of 1MT, L-tryptophan, kynurenine, 3-nitro-L-tyrosine, trichloroacetic acid, PMA and ionomycin were obtained from Sigma Chemicals (St. Louis, MO). FITC-conjugated anti-CD8, anti-IFN-γ, anti-IL-17 and anti-CD14 mAbs, PE-conjugated anti-CD56, anti-CD4, anti-CD62L and IL-4 mAbs, PerCP-conjugated anti-CD45RA mAbs, APC-conjugated anti-CD25 and anti-CD3 mAbs, Cytofix/Cytoperm™ solution and Golgi Plug Protein Transport Inhibitor™ were purchased from BD Biosciences (Mountain View, CA). Rabbit anti-human GAPDH (D16H11), anti-human β-tubulin (9F3), anti-human phosphorylated STAT3 (Tyr705, D3A7) and anti-human STAT3 (79D7) antibodies were from Cell Signaling Technology (Milan, Italy). Horseradish peroxidase (HRP)-conjugated anti-rabbit and anti-mouse secondary antibodies were purchased from Bio-Rad (Hercules, CA). The Human Regulatory T-Cell Staining Kit was obtained from eBioscience (San Diego, CA). KG-1a (a variant sub-line of KG1 AML cells), NB4 [maturation-inducible AML with t(15;17)] and K-562 (chronic myeloid leukemia) cell lines were purchased from LGC Standards (Milan, Italy).

### Cell and serum preparation

BM samples were used to isolate mononuclear cells by density gradient centrifugation on Ficoll-Hypaque (Uppsala, Sweden). Cells were stored in FCS with 10% dimethyl-sulfoxide in the vapor phase of liquid nitrogen until the day of experimental manipulation.

### Intracellular cytokine staining

Cytokine production at the single-cell level was assessed with mAbs directed against IL-4, IL-17 and IFN-γ. CD4^+^ cells were activated for 5 hours with 50 ng/ml PMA and 1 μg/ml ionomycin, in the presence of inhibitors of protein transport. Following fixation and permeabilization, cells were labeled with cytokine-specific mAbs for 30 minutes at 4°C and then analyzed by flow cytometry.

### Real-time quantitative PCR

Total RNA was obtained from cultured AML cells using the RNeasy plus kit (Qiagen, Milan, Italy) according to manufacturer's instructions. Complementary DNA (cDNA) was prepared starting from 1 μg of total RNA using the iScript cDNA Synthesis Kit (Bio-Rad) according to the manufacturer's instructions. Amplifications were carried out using specific primers (IDO1 gene: forward primer 5'→3': GGGACACTTTGCTAAAGGCG; reverse primer 5'→3': GTCTGATAGCTGGGGGTTGC) and the iQ SYBRGreen Supermix (Bio-Rad) in a final volume of 25 μL, starting with a 3-min template denaturation step at 95°C followed by 40 cycles of 15s at 95°C and 1 min at 60°C. β-actin was used as housekeeping gene (forward primer 5'→3': GCCGACAGGATGCAGAAGGAG; reverse primer 5'→3': CAGGATGGAGCCGCCGATC). Standard curves were generated using a serial dilution of the initial amount of control cDNA to determine the range of template concentrations, and showed a good linearity and efficiency for the different reactions. Melt curves of the reaction products were also generated to assess the specificity of the measured fluorescence. Samples were run in triplicate and the mean of threshold cycles (Ct) for each specimen was used to obtain the fold-change of gene expression level, using the following equation: fold change = 2 - (Ct), where Ct = Ct specific gene-Ct β-actin, and (Ct) = Ct specimen-Ct control. Calculations were made with the RelQuant Excel spreadsheet (Bio-Rad).

### ELISA

IFN-γ levels in patient serum or in culture supernatants were quantitated with a commercially available enzyme-linked immunosorbent assay (ELISA; R&D Systems). The limit of detection was 15.6 pg/ml IFN-γ.

### Measurement of Treg cells

Cells were first labeled with anti-CD4 and anti-CD25 mAbs, followed by sequential fixation and permeabilization, and then were stained with Alexa-Fluor 488-conjugated anti-FoxP3 mAb (PCH101 clone), as already detailed [40].

### Western blotting

AML blast cells (6x10^5^) were centrifuged at 1,200 rpm for 10 minutes. Cell pellets were lysed with RIPA buffer [150 mM NaCl, 1% NP-40, 0.5% sodium deoxycholate, 0.1% SDS, 50 mM Tris-HCl (pH=8), 1 mM PMSF, 1 mM EGTA, 50 mM NaF, 50 mM Na3VO4 and protease inhibitors (Roche, Milan, Italy)]. Cell lysates were incubated on ice for 20 minutes and clarified by centrifugation at 14,000 rpm for 20 minutes. Cell extracts were boiled for 5 minutes at 95°C and analyzed by 12% SDS-PAGE. Samples were transferred onto a nitrocellulose membrane (Bio-Rad, Milan, Italy). Blots were probed with primary antibodies at 1:1,000 dilution, washed and developed with horseradish peroxidase-conjugated rabbit or mouse secondary antibodies (Bio-Rad), respectively. A chemiluminescent Western blotting detection reagent was used to reveal the proteins of interest (Amersham, Milan, Italy). Densitometry was used to quantitate any changes in protein expression levels (ImageJ software; National Institutes of Health, Bethesda, MD).

### Immunofluorescence analysis

Cells were run through a FACS Canto II® flow cytometer (BD Biosciences) with standard equipment. A minimum of 30,000 events was collected and acquired in list mode using the FACS Diva® software package (BD Biosciences).

### IDO1 activity

Tryptophan and kynurenine levels were measured with reverse-phase HPLC (Agilent Technologies 1200; Waldbronn, Germany). Briefly, 200 μL sample aliquots were diluted with 200 μL potassium phosphate buffer (0.05 mol/L; pH=6.0) containing 3-nitro-L-tyrosine (100 μmol/L) as internal standard. Proteins were precipitated with 50 μL of 2 mol/L trichloroacetic acid and vials were immediately vortex-mixed and centrifuged for 10 minutes at 13,000g. One-hundred-fifty μL of the supernatants were transferred into micro-vials and placed into the auto-sampling device (Agilent Technologies 1200). Sample were analyzed using a C18HPH ProteCol® HPLC column (SGE Analytical Science, Australia) and a double-pump HPLC apparatus equipped with spectrophotometric and fluorescence detectors (Agilent Technologies). Tryptophan was detected by a fluorescence detector at an excitation wavelength of 285nm and an emission wavelength of 365nm. Kynurenine and nitrotyrosine were detected by recording UV absorbance at a wavelength of 360nm. The concentrations of kynurenine and tryptophan were calculated according to the peak height and were compared both with 3-nitro-L-tyrosine as internal standard and with reference curves constructed with escalating concentrations of L-tryptophan (10 to 30 μmol/L) and kynurenine (10 to 30 μmol/L).

### Co-immunoprecipitation assays

AML blasts were seeded in 6-well plates at 2 x 106/mL and were stimulated with 100 ng/ml IFN-γ for 16 hours. After treatment, cells were harvested, washed with ice-cold PBS and lysed with a Co-IP buffer [137 mM NaCl, 1% NP-40, 20 mM Tris-HCl (pH=8), 10% glycerol, 1 mM PMSF, 1 mM CaCl2, 1 mM MgCl2 50 mM NaF, 1 mM Na3VO4 and Protease Inhibitor Cocktail Tablets (Roche, Milan, Italy)]. Antibodies to IDO1 (primary target antigen) or STAT-3 (interacting protein) were conjugated to Protein A following overnight incubation at 4°C (Pierce; Milan, Italy). The antibody-coupled resin was incubated with cell lysates (2 mg/sample) for 4 hours at 4°C. Immune complexes were boiled with 30 ml of loading buffer (2X) for 5 minutes at 95°C. Total proteins and immune complexes were separated by 10% sodium dodecyl sulphate-polyacrylamide gel electrophoresis (SDS-PAGE) and transferred to nitrocellulose. Membranes were blocked with 5% w/v non-fat dry milk and incubated with appropriate dilutions of anti-STAT3 or anti-IDO1 antibodies, following the manufacturer's instructions. Membranes were probed with horseradish peroxidase (HRP)-conjugated secondary antibodies and were treated with ECL reagent (Euroclone, Milan, Italy). Total cell lysates served as a loading control.

### Statistical methods

The approximation of data distribution to normality was tested preliminarily using statistics for kurtosis and symmetry. Data were presented as median and range, and comparisons were performed with the Mann-Whitney U test for paired or unpaired data, or with the Kruskal-Wallis test with Bonferroni's correction for multiple comparisons, as appropriate. Variables related to leukemia characterization and prognosis (patient age, leukocyte count at diagnosis, FAB classification and molecular genetics) were recorded and incorporated into the final analysis of the results. Overall survival (OS) and event-free survival (EFS) were defined as the probability of survival, regardless of disease status, and of being alive and disease-free at any time point, respectively. The 8-year OS and EFS for children with acute leukemia were calculated with the Kaplan-Meier product limit method, using commercially available software packages. The log-rank test was used to compare differences between groups. *P* values of 0.05 or less denoted statistical significance. The Fisher's exact test was used to compute a *P* value from a contingency table.
